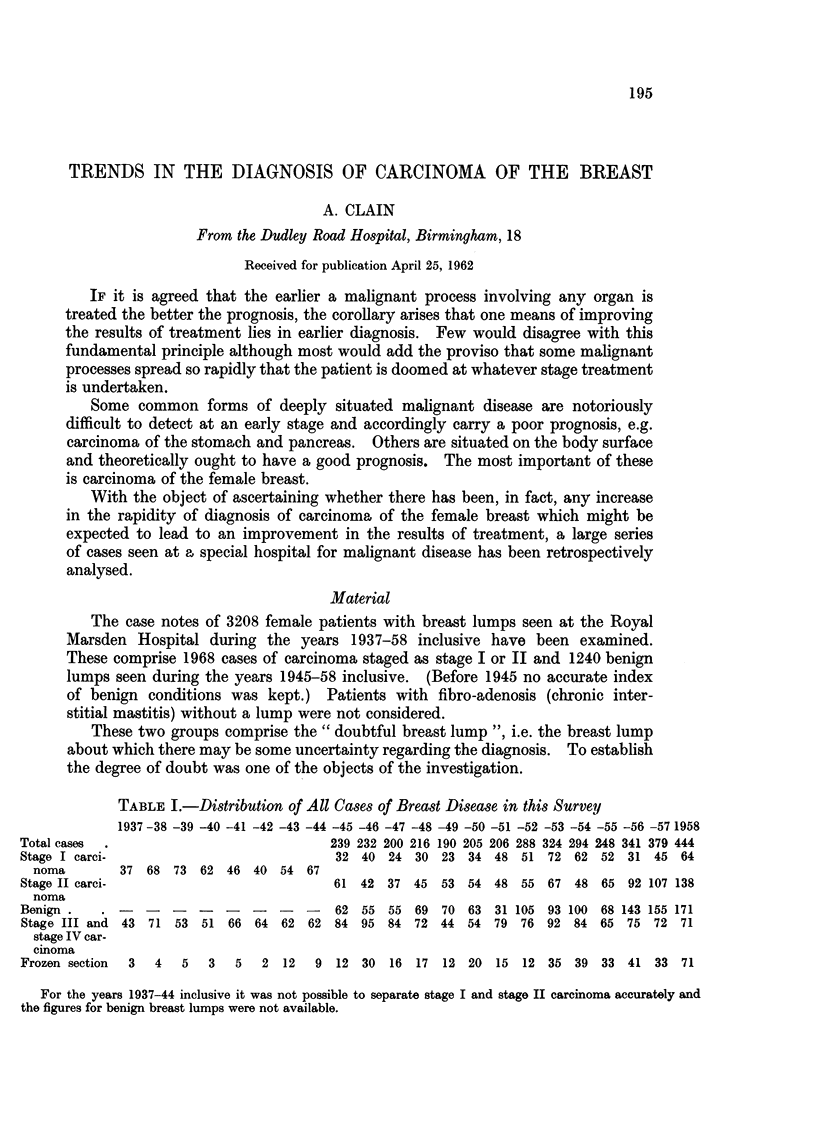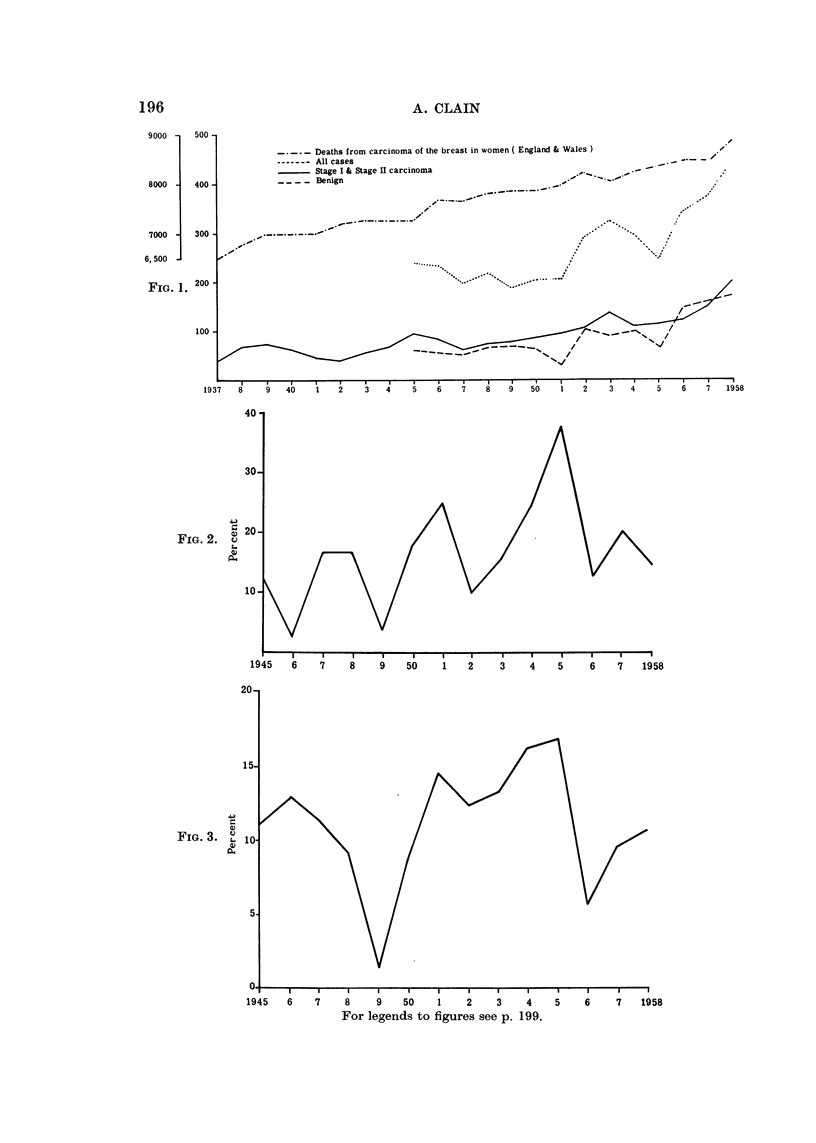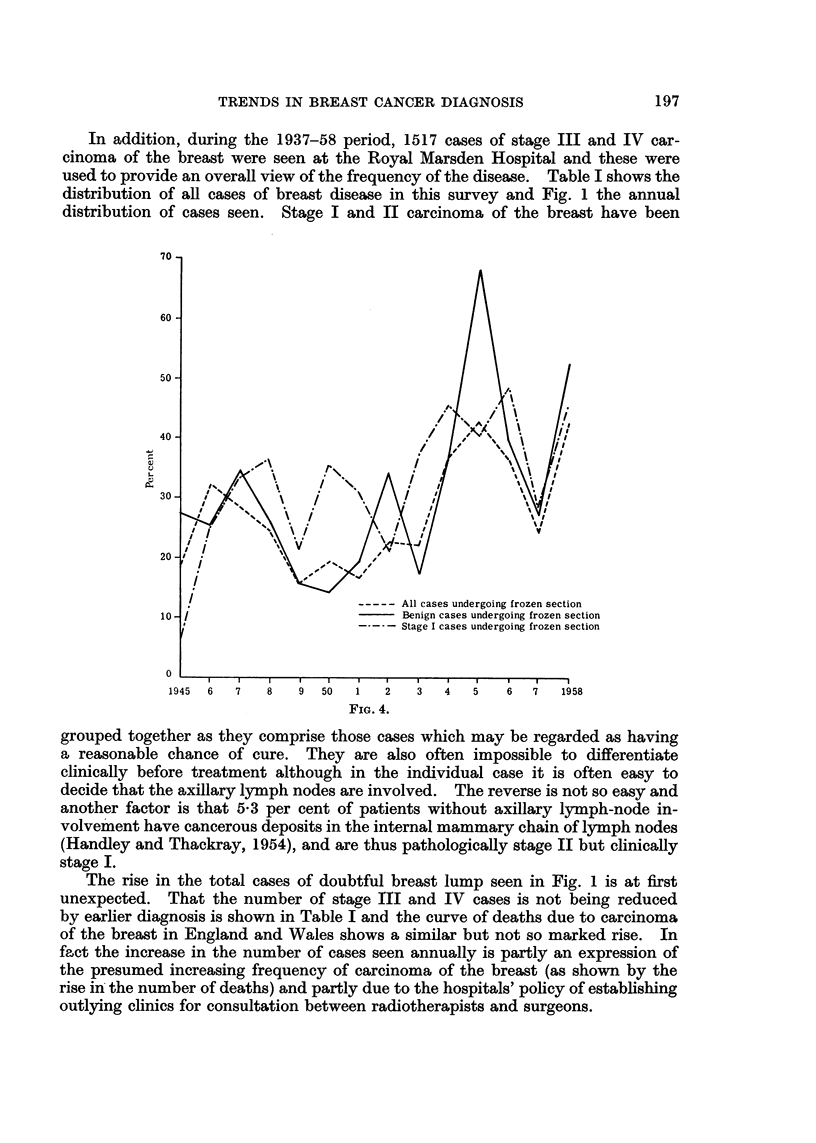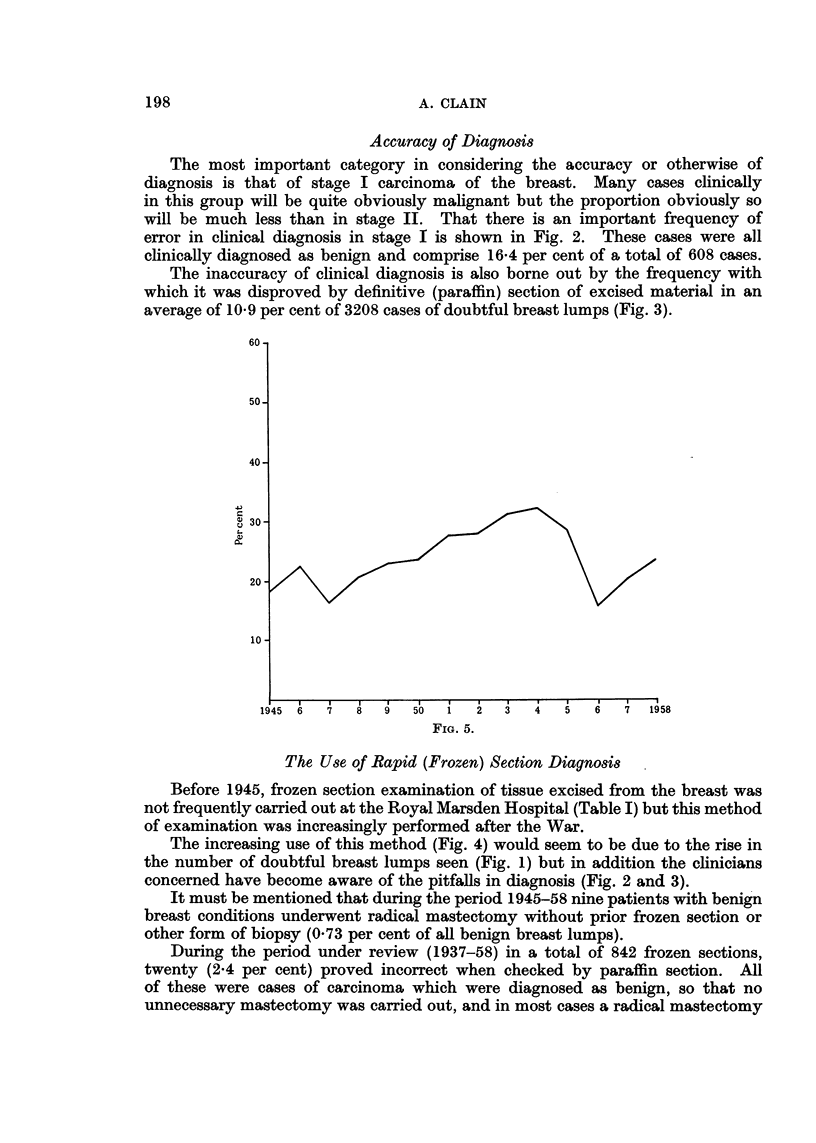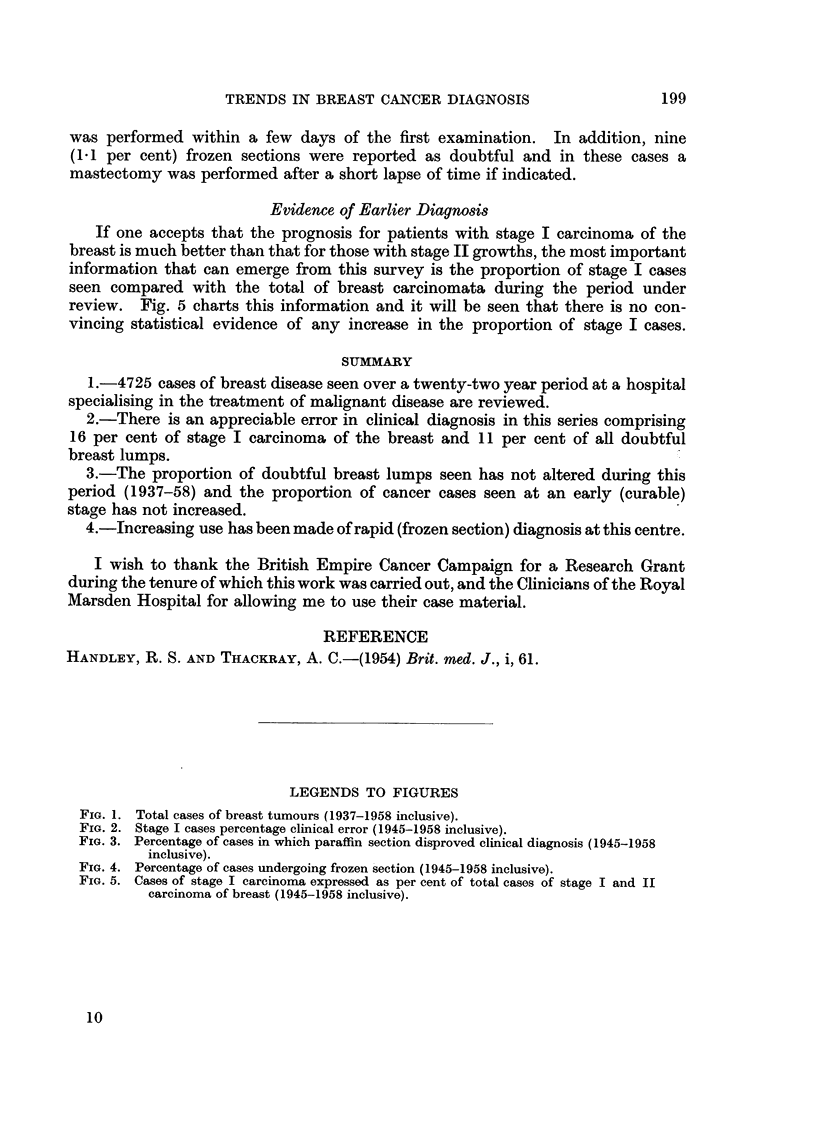# Trends in the Diagnosis of Carcinoma of the Breast

**DOI:** 10.1038/bjc.1962.22

**Published:** 1962-06

**Authors:** A. Clain


### TRENDS IN THE DIAGNOSIS OF CARCINOMA OF THE BREAST

A. CLAIN

*From the Dudley Road Hospital, Birmingham,* 18

Received for publication April 25, 1962

IF it is agreed that the earlier a malignant process involving any organ is treated the better the prognosis, the corollary arises that one means of improving the results of treatment lies in earlier diagnosis. Few would disagree with this fundamental principle although most would add the proviso that some malignant processes spread so rapidly that the patient is doomed at whatever stage treatment is undertaken.

Some common forms of deeply situated malignant disease are notoriously difficult to detect at an early stage and accordingly carry a poor prognosis, e.g. carcinoma of the stomach and pancreas. Others are situated on the body surface and theoretically ought to have a good prognosis. The most important of these is carcinoma of the female breast.

With the object of ascertaining whether there has been, in fact, any increase in the rapidity of diagnosis of carcinoma of the female breast which might be expected to lead to an improvement in the results of treatment, a large series of cases seen at a special hospital for malignant disease has been retrospectively analysed.

### *Material*

The case notes of 3208 female patients with breast lumps seen at the Royal Marsden Hospital during the years 1937–58 inclusive have been examined. These comprise 1968 cases of carcinoma staged as stage I or II and 1240 benign lumps seen during the years 1945–58 inclusive. (Before 1945 no accurate index of benign conditions was kept.) Patients with fibro-adenosis (chronic interstitial mastitis) without a lump were not considered.

These two groups comprise the " doubtful breast lump ", i.e. the breast lump about which there may be some uncertainty regarding the diagnosis. To establish the degree of doubt was one of the objects of the investigation.

TABLE I.—*Distribution of All Cases of Breast Disease in this Survey*

| | 1937 | -38 | -39 | -40 | -41 | -42 | -43 | -44 | -45 | -46 | -47 | -48 | -49 | -50 | -51 | -52 | -53 | -54 | -55 | -56 | -57 | 1958 |
|---|---|---|---|---|---|---|---|---|---|---|---|---|---|---|---|---|---|---|---|---|---|---|
| Total cases | | | | | | | | | 239 | 232 | 200 | 216 | 190 | 205 | 206 | 288 | 324 | 294 | 248 | 341 | 379 | 444 |
| Stage I carcinoma | 37 | 68 | 73 | 62 | 46 | 40 | 54 | 67 | 32 | 40 | 24 | 30 | 23 | 34 | 48 | 51 | 72 | 62 | 52 | 31 | 45 | 64 |
| Stage II carcinoma | | | | | | | | | 61 | 42 | 37 | 45 | 53 | 54 | 48 | 55 | 67 | 48 | 65 | 92 | 107 | 138 |
| Benign | — | — | — | — | — | — | — | — | 62 | 55 | 55 | 69 | 70 | 63 | 31 | 105 | 93 | 100 | 68 | 143 | 155 | 171 |
| Stage III and stage IV carcinoma | 43 | 71 | 53 | 51 | 66 | 64 | 62 | 62 | 84 | 95 | 84 | 72 | 44 | 54 | 79 | 76 | 92 | 84 | 65 | 75 | 72 | 71 |
| Frozen section | 3 | 4 | 5 | 3 | 5 | 2 | 12 | 9 | 12 | 30 | 16 | 17 | 12 | 20 | 15 | 12 | 35 | 39 | 33 | 41 | 33 | 71 |

For the years 1937–44 inclusive it was not possible to separate stage I and stage II carcinoma accurately and the figures for benign breast lumps were not available.

Fig. 1.

Fig. 2.

Fig. 3.

For legends to figures see p. 199.

In addition, during the 1937–58 period, 1517 cases of stage III and IV carcinoma of the breast were seen at the Royal Marsden Hospital and these were used to provide an overall view of the frequency of the disease. Table I shows the distribution of all cases of breast disease in this survey and Fig. 1 the annual distribution of cases seen. Stage I and II carcinoma of the breast have been

FIG. 4.

grouped together as they comprise those cases which may be regarded as having a reasonable chance of cure. They are also often impossible to differentiate clinically before treatment although in the individual case it is often easy to decide that the axillary lymph nodes are involved. The reverse is not so easy and another factor is that 5·3 per cent of patients without axillary lymph-node involvement have cancerous deposits in the internal mammary chain of lymph nodes (Handley and Thackray, 1954), and are thus pathologically stage II but clinically stage I.

The rise in the total cases of doubtful breast lump seen in Fig. 1 is at first unexpected. That the number of stage III and IV cases is not being reduced by earlier diagnosis is shown in Table I and the curve of deaths due to carcinoma of the breast in England and Wales shows a similar but not so marked rise. In fact the increase in the number of cases seen annually is partly an expression of the presumed increasing frequency of carcinoma of the breast (as shown by the rise in the number of deaths) and partly due to the hospitals' policy of establishing outlying clinics for consultation between radiotherapists and surgeons.

*Accuracy of Diagnosis*

The most important category in considering the accuracy or otherwise of diagnosis is that of stage I carcinoma of the breast. Many cases clinically in this group will be quite obviously malignant but the proportion obviously so will be much less than in stage II. That there is an important frequency of error in clinical diagnosis in stage I is shown in Fig. 2. These cases were all clinically diagnosed as benign and comprise 16·4 per cent of a total of 608 cases.

The inaccuracy of clinical diagnosis is also borne out by the frequency with which it was disproved by definitive (paraffin) section of excised material in an average of 10·9 per cent of 3208 cases of doubtful breast lumps (Fig. 3).

FIG. 5.

*The Use of Rapid (Frozen) Section Diagnosis*

Before 1945, frozen section examination of tissue excised from the breast was not frequently carried out at the Royal Marsden Hospital (Table I) but this method of examination was increasingly performed after the War.

The increasing use of this method (Fig. 4) would seem to be due to the rise in the number of doubtful breast lumps seen (Fig. 1) but in addition the clinicians concerned have become aware of the pitfalls in diagnosis (Fig. 2 and 3).

It must be mentioned that during the period 1945–58 nine patients with benign breast conditions underwent radical mastectomy without prior frozen section or other form of biopsy (0·73 per cent of all benign breast lumps).

During the period under review (1937–58) in a total of 842 frozen sections, twenty (2·4 per cent) proved incorrect when checked by paraffin section. All of these were cases of carcinoma which were diagnosed as benign, so that no unnecessary mastectomy was carried out, and in most cases a radical mastectomy was performed within a few days of the first examination. In addition, nine (1·1 per cent) frozen sections were reported as doubtful and in these cases a mastectomy was performed after a short lapse of time if indicated.

### *Evidence of Earlier Diagnosis*

If one accepts that the prognosis for patients with stage I carcinoma of the breast is much better than that for those with stage II growths, the most important information that can emerge from this survey is the proportion of stage I cases seen compared with the total of breast carcinomata during the period under review. Fig. 5 charts this information and it will be seen that there is no convincing statistical evidence of any increase in the proportion of stage I cases.

### SUMMARY

1.—4725 cases of breast disease seen over a twenty-two year period at a hospital specialising in the treatment of malignant disease are reviewed.

2.—There is an appreciable error in clinical diagnosis in this series comprising 16 per cent of stage I carcinoma of the breast and 11 per cent of all doubtful breast lumps.

3.—The proportion of doubtful breast lumps seen has not altered during this period (1937–58) and the proportion of cancer cases seen at an early (curable) stage has not increased.

4.—Increasing use has been made of rapid (frozen section) diagnosis at this centre.

I wish to thank the British Empire Cancer Campaign for a Research Grant during the tenure of which this work was carried out, and the Clinicians of the Royal Marsden Hospital for allowing me to use their case material.

### REFERENCE

HANDLEY, R. S. AND THACKRAY, A. C.—(1954) *Brit. med. J.*, i, 61.

---

### LEGENDS TO FIGURES

FIG. 1. Total cases of breast tumours (1937–1958 inclusive).
FIG. 2. Stage I cases percentage clinical error (1945–1958 inclusive).
FIG. 3. Percentage of cases in which paraffin section disproved clinical diagnosis (1945–1958 inclusive).
FIG. 4. Percentage of cases undergoing frozen section (1945–1958 inclusive).
FIG. 5. Cases of stage I carcinoma expressed as per cent of total cases of stage I and II carcinoma of breast (1945–1958 inclusive).